# Cytochrome P450 CYP1B1 over-expression in primary and metastatic ovarian cancer

**DOI:** 10.1054/bjoc.2001.1907

**Published:** 2001-07

**Authors:** M C E McFadyen, M E Cruickshank, I D Miller, H L McLeod, W T Melvin, N E Haites, D Parkin, G I Murray

**Affiliations:** Departments of Pathology, Obstetrics and Gynaecology, Medicine and Therapeutics, Molecular and Cell Biology, University of Aberdeen, Foresterhill, Aberdeen, AB25 2ZD, UK

**Keywords:** cytochrome P450, docetaxel, anti-cancer drug

## Abstract

Ovarian cancer is the most frequent cause of death from gynaecological malignancies world wide. Little improvement has been made in the long-term outcome of this disease, with the 5-year survival of patients only 30%. This poor prognosis is due to the late presentation of the disease and to the unpredictable response of ovarian cancer to chemotherapy. The cytochrome P450 enzymes are a superfamily of haemoproteins, known to be involved in the metabolic activation and/or detoxification of a number of anti-cancer drugs. CYP1B1 is a tumour-related form of cytochrome P450 which is over expressed in a wide variety of primary tumours of different histological type. The presence of CYP1B1 may be of importance in the modulation of these tumours to anti-cancer drugs. We have conducted a comprehensive immunohistochemical investigation, into the presence of cytochrome P450 CYP1B1 in primary and metastatic ovarian cancer. The key findings of this study are the increased expression of CYP1B1 in the majority of ovarian cancers investigated (92%), with a strong correlation demonstrated between CYP1B1 expression in both primary and metastatic ovarian cancer (*P*= 0.005 Spearman's rank correlation test). In contrast no detectable CYP1B1 was found in normal ovary. © 2001 Cancer Research Campaign http://www.bjcancer.com


*British Journal of Cancer* (2001) **85**(2), 242–246
© 2001 Cancer Research Campaign
doi: 10.1054/ bjoc.2001.1907, available online at http://www.idealibrary.com on IDEAL®

http://www.bjcancer.com

### Cytochrome P450 CYP1B1 over-expression in primary and metastatic ovarian cancer

**MCE McFadyen[1], ME Cruickshank[2], ID Miller[1], HL McLeod[3*], WT Melvin[4], NE Haites[3,4], D Parkin[2] and GI Murray[1]**

Departments of [1]Pathology, [2]Obstetrics and Gynaecology, [3]Medicine and Therapeutics and [4]Molecular and Cell Biology, University of Aberdeen, Foresterhill, Aberdeen, AB25 2ZD UK

**Summary** Ovarian cancer is the most frequent cause of death from gynaecological malignancies world wide. Little improvement has been made in the long-term outcome of this disease, with the 5-year survival of patients only 30%. This poor prognosis is due to the late presentation of the disease and to the unpredictable response of ovarian cancer to chemotherapy. The cytochrome P450 enzymes are a superfamily of haemoproteins, known to be involved in the metabolic activation and/or detoxification of a number of anti-cancer drugs. CYP1B1 is a tumour-related form of cytochrome P450 which is over expressed in a wide variety of primary tumours of different histological type. The presence of CYP1B1 may be of importance in the modulation of these tumours to anti-cancer drugs. We have conducted a comprehensive immunohistochemical investigation, into the presence of cytochrome P450 CYP1B1 in primary and metastatic ovarian cancer. The key findings of this study are the increased expression of CYP1B1 in the majority of ovarian cancers investigated (92%), with a strong correlation demonstrated between CYP1B1 expression in both primary and metastatic ovarian cancer ($P = 0.005$ Spearman's rank correlation test). In contrast no detectable CYP1B1 was found in normal ovary. © 2001 Cancer Research Campaign http://www.bjcancer.com

**Keywords**: cytochrome P450; docetaxel; anti-cancer drug

Ovarian cancer is the leading cause of death from gynaecological malignancies world-wide (Kristensen and Tropé, 1997; Anthoney and Kaye, 1999). Each year in the UK 5000 women present with this disease, with approximately 4000 deaths per annum in the same time period. Definitive local surgery, typically including a total hysterectomy, bilateral salpingo-oophorectomy and omentectomy with or without lymphadenectomy (Qazi and McGuire, 1995) is generally carried out before chemotherapy. Early tumours (stage 1a–1b) have a good prognosis and do not receive chemotherapy.

Adjuvant chemotherapy or radiation is usually given to stage 1c and for most patients with stage 2–4 ovarian cancer. Despite the introduction of platinum-based chemotherapy and the taxanes, little improvement has been made in the long-term outcome of the disease, with the 5-year survival of patients only 30%. Poor prognosis is due to the late presentation of the disease and to the unpredictable response of ovarian cancer to chemotherapy (Lorrigan et al, 1996), as a result of intrinsic drug resistance observed in ovarian cancer (Arts et al, 1999).

The cytochrome P450 enzymes are a superfamily of constitutive and inducible haemoproteins (Nelson et al, 1996) with a central role in the oxidative metabolism of a wide range of xenobiotics including many carcinogens (Shimada and Guengerich, 1991; Gonzalez and Gelboin, 1994). Moreover, a number of cytochrome P450 enzymes are able to influence the response of established tumours to anti-cancer drugs through the activation and/or deactivation of chemotherapeutic agents such as cyclophosphamide, paclitaxel and docetaxel, used to treat a variety of cancers including ovarian (Le Blanc and Waxman, 1989; Sonnischen and Relling, 1994; Kivisto et al, 1995; Marre et al, 1996; Iyer and Ratain, 1998).

We have previously shown that several cytochrome P450 enzymes (CYP1A, CYP1B1, CYP2C and CYP3A) are over-expressed in a variety of tumours (Murray et al, 1993, 1994, 1997; McKay et al, 1993; Murray 2000). In particular CYP1B1 appears to be a tumour-related form which is over-expressed in a number of primary tumours (Murray et al, 1997, 2001) but is undetectable in normal tissues especially liver. We have shown strong CYP1B1 immunoreactivity in a small number of primary serous cystadenocarcinomas the most common histological subtype of ovarian cancer (Murray et al, 1997). However, its presence in metastatic tumours has not previously been investigated.

We have recently shown that CYP1B1 inactivates docetaxel and our in vitro studies have shown that the presence of CYP1B1 in cells exposed to docetaxel increases the resistance of those cells to the cytotoxic effects of this anti-cancer drug (McFadyen et al, 2001; Rochat et al, 2001). The findings that CYP1B1 may inactivate docetaxel, a drug currently used as second line treatment of ovarian cancer, and which is currently being evaluated for licensing as first line treatment of ovarian cancer, also necessitated a clearer understanding of the presence of CYP1B1 in the different histological subtypes of both primary and metastatic ovarian cancer. This study investigated the presence of CYP1B1 in both primary and metastatic ovarian cancer.

*Received 20 November 2000*
*Revised 23 April 2001*
*Accepted 30 April 2001*

*Correspondence to:* MCE McFadyen

*Current address: Washington University, School of Medicine, St Louis MO 63110-1093 USA

**Table 1** Clinicopathological characteristics of patients with ovarian cancer

| Age (median and range) | 63 years (30–89 years) |
|---|---|
| Stage of ovarian cancer | |
| 1 | 44/172 (25%) |
| 2 | 15/172 (9%) |
| 3 | 103/172 (60%) |
| 4 | 10/172 (6%) |
| Histological subtype | |
| Serous cystadenocarcinoma | 102/172 (59%) |
| Endometrioid carcinoma | 35/172 (21%) |
| Mucinous cystadenocarcinoma | 24/172 (14%) |
| Clear cell adenocarcinoma | 7/172 (4%) |
| Malignant mixed Mullerian tumour | 4/172 (2%) |

**Table 2** Therapeutic regimen of patients with ovarian cancer

| Treatment | Number of patients |
|---|---|
| Carboplatin | 56 (32%) |
| Cisplatin+cyclophosphamide | 32 (19%) |
| None | 30 (17%) |
| Carboplatin+docetaxel | 15 (9%) |
| Carboplatin+paclitaxel | 15 (9%) |
| Cisplatin+paclitaxel | 9 (5%) |
| Treosulphan | 7 (4%) |
| Cisplatin+docetaxel | 4 (2%) |
| No information available | 2 (1%) |
| XRT (radiotherapy) | 1 (1%) |
| Docetaxel | 1 (1%) |
| Total | 172 (100%) |

### MATERIALS AND METHODS

### Tissue

Samples of ovarian cancer and corresponding normal ovary submitted to the Department of Pathology, University of Aberdeen for diagnosis, over a 5-year period (1993–1998), were used in this study. Samples of primary ovarian cancer were available from 167 patients. Of these, 43 patients had corresponding synchronous omental metastases, (i.e. both primary and metastatic tumour available for study). In addition a further 5 cases from patients with metastatic tumours were available which had no corresponding primary tumour (i.e. a total of 172 cases 167 + 5 = 172). In 49 out of the 167 patients with primary ovarian cancer contralateral normal ovarian tissue was also available for study. All the tissue samples had been fixed in 10% neutral buffered formalin for 24 hours and then routinely processed to paraffin wax.

The diagnosis of ovarian cancer was performed with haematoxylin and eosin stained sections using standard histopathological criteria. All tumours were reviewed by a specialist gynaecological pathologist (IDM). The tumours were classified according to criteria described by FIGO (Federation Internationale de Gynecologie et d'Obstetrique). The median age of patients in this study was 63 years with an age range from 30–89 years (Table 1). Of the 172 patients used in this study information on treatment was available for 170 and details are listed in Table 2. The following anti-cancer drugs (cisplatin, carboplatin, cyclophosphamide, paclitaxel, and docetaxel) were used to treat the patients. Most patients received either cisplatin or carboplatin. The other 3 drugs were usually given in combination in a platinum-based regime.

Following diagnosis, the disease status of patients was assessed by 2 gynaecological oncologists as follows, at 3 monthly intervals for the first year, at 6 monthly intervals for the following 2 years, and thereafter. If patients had stable disease or were disease-free, they were seen at yearly intervals. Following surgical debulking only 19% of patients remained disease-free over the period of follow-up, 42% had recurrent disease with a median disease-free interval of 23 months and an overall disease-free range of 1 to + 70 months, 39% of the patients had incomplete tumour debulking at surgery and showed no disease-free interval. The median overall survival of the patients was 17 months with an overall survival range of 1 to at least +70 months.

### Immunohistochemistry

In this study a monoclonal antibody (MAb) raised against a 15 amino acid peptide located in the C-terminal third of the human CYP1B1 protein (McFadyen et al, 1999) was used to identify sites of CYP1B1 immunoreactivity. Immunohistochemical detection of CYP1B1 was performed using a peroxidase-based tyramine signal amplification method as described previously (McFadyen et al, 1999).

Sections of normal ovary and ovarian cancer were dewaxed, rehydrated and washed in cold water, prior to heat-based antigen retrieval in 0.01 M citrate buffer, pH 6 for 20 minutes. The sections were then immunostained with the anti-CYP1B1 monoclonal antibody. Sites of CYP1B1 immunoreactivity were identified using a fluorescein tyramide technique (NEN, Hounslow, Middlesex, UK) with diaminobenzidine as the enzyme substrate. Sections of breast cancer which had previously been shown to contain CYP1B1 by immunohistochemistry (McFadyen et al, 1999) were used as the positive control. The negative control used TBS in place of the primary antibody.

To establish the presence or absence of CYP1B1 and its distribution, intensity and cellular localization, the sections were counterstained with haemotoxylin and examined using bright field light microscopy by 2 independent observers (MCEM, GIM). CYP1B1 immunoreactivity in the tumours was assessed as either strong (3), moderate (2), weak (1) or negative (0). Tumours exhibiting CYP1B1 immunoreactivity in more than 5% of the cells were considered as positive. A tumour was classified as negative if less than 5% of tumour cells showed positive immunoreactivity (McFadyen et al, 1999).

### Statistics

Statistical analysis including survival analysis was performed using SPSS version 7.5 for Windows 95™. The comparison between CYP1B1 expression in primary and metastatic ovarian cancer was performed using the Spearman's rank correlation test. Comparison between survival, CYP1B1 status and the different chemotherapeutic treatments was performed with the Kaplan–Meier log rank test.

### RESULTS

### Immunohistochemistry

#### *Primary ovarian cancer*

CYP1B1 immunoreactivity was identified in the majority (92%) of ovarian cancers and was specifically localized to the cytoplasm of

*© 2001 Cancer Research Campaign*

tumour cells (Figure 1). In addition, there was no apparent intratumour heterogeneity. In a high percentage of the ovarian cancers there was moderate (23%) or strong (51%) immunoreactivity for CYP1B1, while in a minority of cases (17%) there was weak immunostaining for CYP1B1 only 8% of ovarian cancers showed no CYP1B1 immunostaining (Table 3). The different stages of ovarian cancer all exhibited a high level of CYP1B1 expression: stage 1, 91%; stage 2, 73%; stage 3, 95% and stage 4, 90%. A high level of expression was also observed for the different histological subtypes, serous 92%; mucinous 87%; endometrioid 94%; mixed malignant Mullerian 100% and clear cell 86%.

### *Metastatic ovarian cancer*

The presence of CYP1B1 was observed in the majority (94%) of metastases with a high proportion showing moderate (46%) or strong (38%) immunoreactivity, weak (10%) immunoreactivity or absence (6%) of immunoreactivity was found in a minority of cases (Table 4). There was no detectable CYP1B1 expression in any of the normal ovarian tissue samples (Figure 1).

Similar levels of CYP1B1 expression were exhibited for the different histological subtypes in both primary and metastatic tumour (serous, mucinous endometrioid and clear cell) (Tables 3 and 4). The comparison between CYP1B1 expression in primary ovarian cancer and metastatic deposits showed a significant correlation ($P = 0.005$ Spearman's rank correlation test) (Table 5).

In this investigation there was no significant difference in survival of patients on different chemotherapeutic treatments. In addition, the presence of CYP1B1 generally had no effect on the survival of patients on the different treatments. However, in the small group of patients treated with docetaxel either singly or in combination with a platinum-based agent ($n = 20$), a trend towards a poorer overall survival was observed, in those patients with moderate or strong CYP1B1 immunoreactivity (Kaplan–Meier log rank test $P = 0.2716$) (Figure 2).

### DISCUSSION

This study has investigated the presence of CYP1B1 in a large number of both primary and metastatic ovarian tumours, utilizing a monoclonal antibody to CYP1B1 to demonstrate the localization of CYP1B1 to ovarian tumour cells and lack of expression in normal ovarian tissue. The over-expression of CYP1B1 was observed in all the histological subtypes of epithelial ovarian cancer and at high frequency. This is in agreement with a pilot study which utilized a polyclonal antibody to CYP1B1 (Murray et al, 1997), and showed the over-expression of CYP1B1 in a small number ($n = 7$) of serous cystadenocarcinomas investigated. This is the most common histological subtype found in patients suffering from this malignancy. We have previously shown the over-expression of CYP1B1 protein in a wide variety of primary malignant tumours of different histogenetic types and the lack of detectable CYP1B1 protein in normal tissue (Murray et al, 1997). However, no information is available on the presence of CYP1B1 in metastatic disease. It is important to determine the phenotype of the metastatic disease as it may not reflect that of the primary tumour.

Due to the lack of available tissue from metastatic deposits, tissue from the primary tumour is often used to predict the response of the metastatic tumour to chemotherapy (McLeod et al, 2000). This may not be a true reflection of the biological phenotype of the metastatic tumour, indeed differential expression of several proteins has previously been observed between primary and metastatic tumours (McKay et al, 2000). These differences in expression are likely to affect the response of metastatic lesions to chemotherapy. Spread of ovarian cancer is through the peritoneal cavity and one of the key findings of this current study is the high frequency of CYP1B1 over-expression observed in the majority of metastatic tumours. Indeed this study is the first to describe the over-expression of CYP1B1 in metastatic tumours. A highly significant correlation of CYP1B1 immunoreactivity was observed

**Table 3** The number of cases showing CYP1B1 expression in the different histological types of primary ovarian cancer

| Diagnosis | CYP1B1 immunoreactivity | | | | |
|---|---|---|---|---|---|
| | Negative | Weak | Moderate | Strong | Total |
| Serous cystadenocarcinoma | 8 | 15 | 22 | 54 | 99 |
| Endometrioid carcinoma | 2 | 7 | 13 | 13 | 35 |
| Mucinous cystadenocarcinoma | 3 | 4 | 4 | 12 | 23 |
| Clear cell adenocarcinoma | 1 | 3 | 0 | 3 | 7 |
| Malignant mixed Mullerian tumour | 0 | 0 | 0 | 3 | 3 |
| Total | 14 | 29 | 39 | 85 | 167 |

**Table 4** The number of cases of CYP1B1 expression in the different histological types of metastatic ovarian cancer

| Diagnosis | CYP1B1 immunoreactivity | | | | |
|---|---|---|---|---|---|
| | Negative | Weak | Moderate | Strong | Total |
| Serous cystadenocarcinoma | 2 | 4 | 20 | 13 | 39 |
| Endometrioid carcinoma | 0 | 1 | 0 | 4 | 5 |
| Mucinous cystadenocarcinoma | 0 | 0 | 2 | 0 | 2 |
| Malignant mixed Mullerian tumour | 1 | 0 | 0 | 1 | 2 |
| Total | 3 | 5 | 22 | 18 | 48* |

*48 cases comprised of 43 cases with omental metastases and corresponding primary tumour plus 5 cases with metastatic disease but no corresponding primary tumour.

 *© 2001 Cancer Research Campaign*

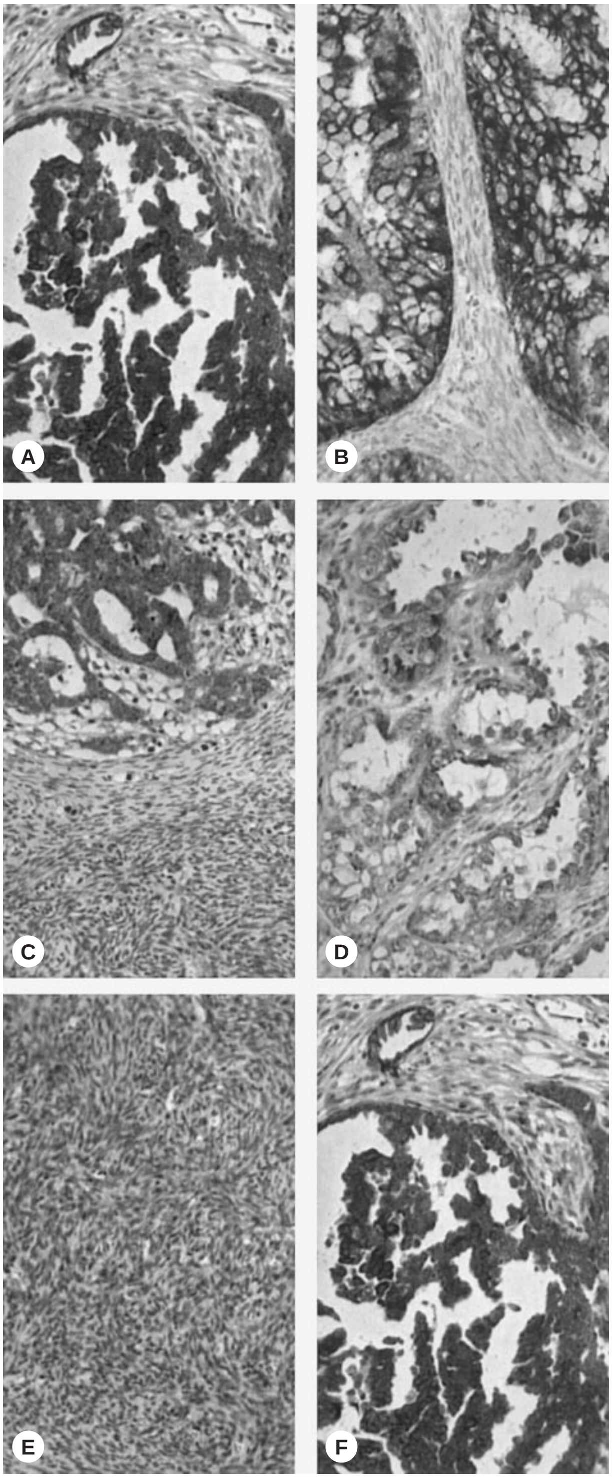

**Figure 1** The immunohistochemical localization of CYP1B1 in different histological subtypes of ovarian cancer and in metastatic deposits. CYP1B1 immunoreactivity is localized to tumour cells. CYP1B1 immunoreactivity is present in both primary tumour and in metastatic deposits and is absent in normal ovary. (**A**) Serous cystadenocarcinoma; (**B**) mucinous cystadenocarcinoma; (**C**) endometrioid carcinoma; (**D**) clear cell adenocarcinoma; (**E**) normal ovary; (**F**) metastatic serous cystadenocarcinoma between the primary and metastatic tumours ($P$ = 0.005 Spearman's rank correlation test).

**Table 5** Comparison of CYP1B1 immunoreactivity in paired samples of primary and metastatic ovarian cancer

| | CYP1B1 in primary tumour | | | | |
|---|---|---|---|---|---|
| | **Negative** | **Weak** | **Moderate** | **Strong** | **Total** |
| Negative | 1 | 0 | 0 | 2 | 3 |
| Weak | 0 | 1 | 2 | 1 | 4 |
| Moderate | 1 | 3 | 5 | 10 | 19 |
| Strong | 0 | 0 | 3 | 14 | 17 |
| Total | 2 | 4 | 10 | 27 | 43 |

$P$ = 0.005 Spearman's rank correlation test.

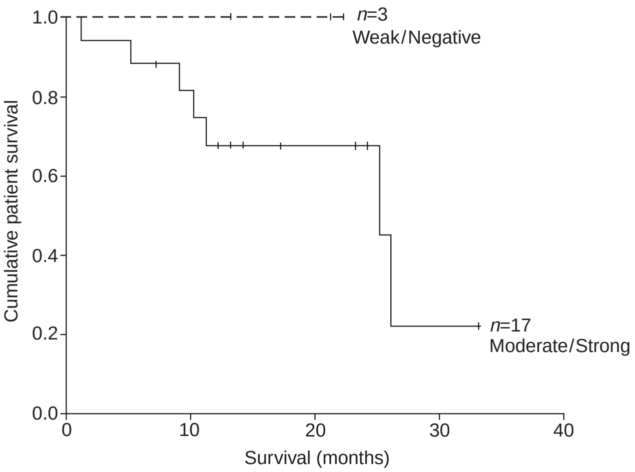

**Figure 2** Survival of patients treated with docetaxel as part of their anti-cancer drug regimen and classified according to CYP1B1 status of their primary tumours. The presence of strong/moderate CYP1B1 immunoreactivity results in a poorer overall survival compared with weak or absent CYP1B1 immunoreactivity (Kaplan–Meier log rank test $P$ = 0.2716)

In this study a trend suggesting a poorer survival was shown in those patients with moderate or strong CYP1B1 immunoreactivity who received docetaxel either as a single agent or in combination with a platinum-based agent, compared to those patients with low or negative CYP1B1 immunoreactivity. Although, this finding was not statistically significant (Kaplan–Meier log rank test $P$ = 0.2716), this may be due to the small number of patients treated with docetaxel. However, this finding supports our in vitro studies and the concept that CYP1B1 inactivates docetaxel and increases the resistance of cells expressing CYP1B1 to the cytotoxic effects of this anti-cancer drug (McFadyen et al, 2001; Rochat 2001). Therefore, further investigation with a larger population is required to ascertain whether CYP1B1 does contribute significantly to the response of ovarian cancer to docetaxel.

We have recently shown that CYP1B1 interacts with a number of structurally diverse clinically useful anti-cancer agents in a substrate-dependent manner (Rochat et al, 2001). Therefore, in addition to being a mechanism of docetaxel drug resistance it is likely that CYP1B1 is a mechanism of drug resistance to a variety of anti-cancer drugs. The findings that CYP1B1 immunoreactivity was over-expressed in the majority of primary and metastatic tumours, and the lack of detectable CYP1B1 in normal tissue including the ovary, also highlights CYP1B1 as a target for the development of novel chemotherapeutic agents which could be activated by this P450 (Murray et al, 1997; McFadyen et al, 1999).

*© 2001 Cancer Research Campaign*

### ACKNOWLEDGEMENT

This research was funded by the Chief Scientist Office Scottish Health Department.

### REFERENCES

Anthoney DA and Kaye SB (1999) Epithelial ovarian cancer. *Presc J* **39**: 65–71
Arts HJ, Katsaros D, de Vries EG, Massobrio M, Genta F, Danese S, Arisio R, Scheper RJ, Kool M, Scheffer G1, Willemse PH, van der Zee AG and Suurmeijer AJ (1999) Drug resistance-associated markers P-glycoprotein, multidrug resistance-associated protein 1, multidrug resistance-associated protein 2 and lung resistance associated protein as prognostic factors in ovarian carcinoma. *Clin Cancer Res* **5**: 2798–2805
Gonzalez FJ and Gelboin HV (1994) Role of human cytochromes P450 in the metabolic activation of chemical carcinogens and toxins. *Drug Metab Rev* **26**: 165–183
Hayes CL, Spink DC, Spink BC, Cao JQ, Walker NJ and Sutter TR (1996) 17-β-Estradiol hydroxylation catalysed by human cytochrome P450 1B1. *Proc Natl Acad Sci USA* **93**: 9776–9781
Iyer L and Ratain MJ (1998) Pharmacogenetics and cancer chemotherapy. *Eur J Cancer* **34**: 1493–1499
Kivisto KT, Kroemer HK and Eichelbaum M (1995) The role of human cytochrome P450 enzymes in the metabolism of anti-cancer agents: implications for drug interactions. *Br J Clin Pharmacol* **40**: 523–530
Kristensen GB and Tropé C (1997) Epithelial ovarian carcinoma. *Lancet* **349**: 113–117
Le Blanc GA and Waxman DJ (1989) Interaction of anticancer drugs with hepatic monooxygenase enzymes. *Drug Metab Rev* **20**: 395–439
Lorrigan PC, Crosby T and Coleman RE (1996) Current drug treatment guidelines for epithelial ovarian cancer. *Drugs* **51**: 571–584
Marre F, Sanderink GJ, de Sousa G, Gaillard C, Martinet M and Rahmani R (1996) Hepatic biotransformation of docetaxel (Taxotere) in vitro: involvement of the CYP3A subfamily in humans. *Cancer Res* **56**: 1296–1302
McFadyen MCE, Breeman S, Payne S, Stirk C, Miller ID, Melvin WT and Murray GI (1999) Immunohistochemical localisation of cytochrome P450 CYP1B1 in breast cancer with monoclonal antibodies specific for CYP1B1. *J Histochem Cytochem* **47**: 1457–1464
McFadyen MCE, McLeod HL, Jackson FC, Melvin WT, Doehmer J and Murray GI (2001) Cytochrome P450 CYP1B1 protein expression: a novel mechanism of anti-cancer drug resistance. *Biochemical Pharmacology* **62**: 207–212
McKay JA, Murray GI, Weaver RJ, Ewen SW, Melvin WT and Burke MD (1993) Xenobiotic metabolising enzyme expression in colonic neoplasia. *Gut* **34**: 1234–1239
McKay JA, Douglas JJ, Ross VG, Curran S, Ahmed FY, Loane JF, Murray GI and McLeod HL (2000) Expression of cell cycle proteins in primary colerectal tumours does not always predict expression in lymph node matastases. *Clin Cancer Res* **6**: 1113–1118
McLeod HL, McKay JA, Collie-Duguid ESR and Cassidy J (2000) Therapeutic opportunities from tumour biology in metastatic colon cancer. *Eur J Cancer* **36**: 1706–1712
Murray GI (2000) The role of cytochrome P450 in tumour development and progression and its potential in therapy. *J Pathol* **192**: 419–426
Murray GI, McKay JA, Weaver RJ, Ewen SW, Melvin WT and Burke MD (1993) Cytochrome P450 expression is a common molecular event in soft tissue sarcomas. *J Pathol* **171**: 49–52
Murray GI, Shaw D, Weaver RJ, McKay JA, Ewen SW, Melvin WT and Burke MD (1994) Cytochrome P450 expression in oesophageal cancer. *Gut* **35**: 599–603
Murray GI, Taylor MC, McFadyen MCE, McKay JA, Greenlee WF, Burke MD and Melvin WT (1997) Tumour specific expression of cytochrome P450 CYP1B1. *Cancer Res* **57**: 3026–3031
Murray GI, Melvin WT, Greenlee WF and Burke MD (2001) Regulation, function and tissue-specific expression of cytochrome P450 CYP1B1. *Annu Rev Pharmacol Toxicol* **41**: 297–316
Nelson DR, Koymans L, Kamataki T, Stegman JJ, Feyeteisen R, Waxman DJ, Waterman MR, Gotoh O, Coon MJ, Estabrook RW, Gunsalas IC and Nebert DW (1996) P450 superfamily: update on new sequences, gene mapping, accession numbers and nomenclture. *Pharmacogenetics* **6**: 1–42
Quazi F and McGuire WP (1995) The treatment of epithelial ovarian cancer. *CA-A Cancer J Clin* **45**: 88–101
Rochat B, Morsman JM, Murray GI, Figg WD and McLeod HL (2000) Human CYP1B1 and anti-cancer agent metabolism: mechanism for tumour specific drug interaction? *J Pharmacol Exp Ther* **296**: 537–541
Shimada T and Guengerich FP (1991) Oxidation of toxic and carcinogenic chemicals by human cytochrome P450 enzymes. *Chem Res Toxicol* **4**: 391–407
Sonnichsen DS and Relling MV (1994) Clinical pharmacokinetics of paclitaxel. *Clin Pharmacokinet* **27**: 256–269

© 2001 Cancer Research Campaign
